# Decreased nutrient digestibility due to viscosity is independent of the amount of dietary fibre fed to growing pigs

**DOI:** 10.1017/S0007114521000866

**Published:** 2022-01-28

**Authors:** Yuan-Tai Hung, Jinlong Zhu, Gerald C. Shurson, Pedro E. Urriola, Milena Saqui-Salces

**Affiliations:** 1 Department of Animal Science, University of Minnesota, 1988 Fitch Ave., St. Paul, MN 55108, USA; 2 Department of Veterinary Population Medicine, University of Minnesota, 1365 Gortner Ave., St. Paul, MN 55108, USA

**Keywords:** Dietary fibre functionality, Nutrient utilisation, Digestive physiology, Monogastric animals

## Abstract

Fibre content and its effect on chyme viscosity are associated with changes in the digestive system of humans and pigs. It is unclear if fibre content and viscosity affect digestive function independently or interactively. We evaluated apparent ileal digestibility (AID) of nutrients and intestinal function in thirty-six ileal-cannulated barrows fed for 29 d either maize–soyabean meal (MSBM) or high-fibre MSBM + 30 % distillers dried grains with solubles (MSBM + DDGS) modified to three levels of viscosity by adding 5 % non-viscous cellulose (CEL), 6·5 % medium-viscous carboxymethylcellulose (MCMC) or 6·5 % high-viscous CMC (HCMC). Digesta were collected on days 27 and 28 and intestinal samples on day 29. Feeding CMC, regardless of fibre content, increased viscosity of whole digesta (*P* = 0·003) and digesta supernatant (*P* < 0·0001) compared with CEL. Feeding MSBM + DDGS or CMC decreased AID of DM (*P* = 0·003; *P* < 0·0001) and crude protein (*P* = 0·02; *P* < 0·0001) compared with MSBM or CEL. Feeding CMC regardless of fibre content increased jejunal crypt depth (*P* = 0·02) and ileal goblet cell area (*P* = 0·004) compared with CEL. Adding DDGS or CMC did not affect villus height and gene expression of jejunal monosaccharide and amino acid transporters. Feeding HCMC, regardless of fibre content, elevated amylase activity by 46 and 50 % in jejunal (*P* = 0·03) and ileal digesta (*P* = 0·01) compared with CEL. In summary, diets with increased viscosity decreased nutrient digestibility and induced intestinal changes that were independent of the amount of fibre fed.

Dietary fibre (DF) intake has been associated with health benefits such as lowering risk for obesity, metabolic diseases, cancers, chronic diseases and supporting gut health^(1,2)^. Continuous consumption of DF is important for maintenance of gut mucosal integrity, supporting a favourable microbiome and improving immune response for humans and animals^(1,3,4)^. In addition, there is increasing interest for incorporating various DF-rich alternative feedstuffs to swine for optimising intestinal function and health status, as well as reducing diet costs without compromising growth performance^(3–5)^. In pigs, typical rations are maize and soyabean meal-based diets that contain about 8 % neutral-detergent fibre (NDF), whereas the inclusion of DF-rich ingredients can elevate dietary NDF to about 20 %, depending on the inclusion rate and composition of ingredients^(6,7)^. Feeding high-fibre diets (e.g. 20 % NDF) can induce intestinal adaptation characterised by changes in intestinal cell turnover, morphology^(8)^ and increased goblet cells^(6,7)^ compared with typical maize and soyabean meal-based diets. Overall, a common effect of including high levels of DF in human and pig diets is the reduction of nutrient digestibility^(4,9,10)^.

DF is often analysed through measuring crude fibre, acid or neutral-detergent and total DF methods, which quantify indigestible carbohydrates in feedstuffs. These analytical methods have limited capability to characterise the physiological effects of DF. The physical–chemical properties of DF – fermentability, solubility and viscosity – provide useful characterisation of nutritional and physiological responses to DF^(11,12)^. Research has shown that viscosity and fermentability of DF are factors that modulate digestive physiology by affecting digesta passage rate, energy and nutrient digestibility^(13)^, and fermentation kinetics that affect the production of SCFA in pigs^(14)^. Viscosity likely plays a role in influencing small intestine digestion and absorption function, metabolism of nutrients and production performance in animals^(5,11,12,15–17)^. Viscosity may also affect microbial fermentation in the large intestine, affecting an important source of energy for colonic cells, although not the major contributor to satisfy energy need of pigs^(3)^. It is estimated that energy produced from SCFA contributes up to 15 % of the maintenance energy requirements of growing pigs^(18)^. In the present study, we have focused on the function of the small intestine. Although both fibre content and viscosity are likely important for gastrointestinal physiological responses to diet, there is a dearth of information regarding which of these factors plays a dominant role and whether they interact in influencing small intestinal function.

In the present study, we focused on evaluating the roles of DF content and viscosity on changes in nutrient digestibility and intestinal responses. We hypothesised that increased viscosity would cause greater effects on nutrient digestibility and changes on intestinal physiology than the DF content. In order to increase DF content and viscosity independently, maize distillers dried grains with solubles (DDGS) and carboxymethylcellulose (CMC), a viscous and non-fermentable polysaccharide, were used in the experimental diets to change DF content and viscosity, respectively. These dietary treatments allowed us to evaluate the effect of DF content and viscosity on nutrient digestibility, intestinal morphology, expression of nutrient transporters and digestive enzyme activities.

## Materials and methods

The Institutional Animal Care and Use Committee at the University of Minnesota reviewed and approved the animal use protocol (#1703-34701A) for the present study.

### Animals, diets and experimental design

A total of thirty-six barrows (initial body weight (BW) = 26·5 (3·9) kg) from Topigs females (Landrace × Yorkshire) sired by Duroc boars (Compart’s Boar Store Inc.) were housed individually in metabolism crates equipped with a stainless steel feeder and a nipple drinker at the UMN Southern Research and Outreach Centre. Pigs were fitted with a T-cannula with an inner diameter of 1·6 cm at approximately 10 cm from the ileocecal valve. After 14 d post-surgery, pigs were allotted to six blocks of six pigs with similar initial BW. Within each block, pigs were assigned to one of six dietary treatments in a 2 × 3 factorial arrangement with two basal diets – maize–soyabean meal (MSBM) and MSBM + 30 % DDGS diets and three levels of viscosity: non-viscous cellulose (CEL) at 5 % inclusion, medium-viscous CMC (MCMC) at 6·5 % inclusion and high-viscous CMC (HCMC) at 6·5 % inclusion (Table 1). Cellulose (Ticalose 100 cellulose powder) and CMC (Ticalose^®^ CMC 6000 and 15000) were purchased from TIC Gums (White Marsh). Medium- and high-viscous CMC had a minimum viscosity of 4000 and 7500 mPa·s, respectively. All diets were formulated to meet or exceed the nutritional requirements of growing pigs according to the National Research Council nutrient requirements of swine^(19)^. Titanium dioxide (TiO_2_) was included at 0·5 % as an indigestible marker in diets for calculation of digestibility. Daily feed allowance was calculated based on 3 × maintenance energy requirement of growing-finishing pigs (824·25 × kJ/kg BW^0·60^) and was fed in two equal meals at 0800 h and 1600 h. All pigs were provided *ad libitum* access to water from nipple drinkers throughout the experiment.

**Table 1.** Ingredient and nutrient composition of experimental diets (as-fed basis)*

Fibre content	MSBM			DDGS		
Viscosity	CEL	MCMC	HCMC	CEL	MCMC	HCMC
Ingredient composition, %						
Maize, yellow dent	54·80	52·51	52·51	29·72	27·88	27·88
Soyabean meal	33·77	34·36	34·36	29·36	29·51	29·51
Maize DDGS	–	–	–	30·00	30·00	30·00
Soyabean oil	3·47	4·17	4·17	3·24	3·93	3·93
Monocalcium phosphate	1·08	1·08	1·08	0·54	0·54	0·54
Limestone	0·88	0·88	0·88	1·14	1·13	1·13
Salt	0·25	–	–	0·25	–	–
Premix†	0·25	0·25	0·25	0·25	0·25	0·25
Titanium dioxide	0·50	0·50	0·50	0·50	0·50	0·50
CEL‡	5·00	–	–	5·00	–	–
MCMC§	–	6·25	–	–	6·25	–
HCMC||	–	–	6·25	–	–	6·25
Calculated nutrient composition						
ME, MJ/kg	13·81	13·81	13·81	13·81	13·81	13·81
Crude protein	20·25	20·33	20·33	24·34	24·25	24·25
Standardised ileal digestible (SID) Lys, %	1·0	1·01	1·01	0·98	0·98	0·98
SID Thr, %	0·66	0·58	0·58	0·73	0·73	0·73
SID Met + Cys, %	0·58	0·58	0·58	0·68	0·68	0·68
SID Trp, %	0·22	0·22	0·22	0·22	0·22	0·22
Acid-detergent fibre, %	8·70	9·92	9·92	11·37	12·58	12·58
Neutral-detergent fibre, %	13·70	14·80	14·80	20·00	21·09	21·09
Standardised total tract digestible *P*, %	0·30	0·30	0·30	0·33	0·33	0·33
Analysed nutrient composition, %						
DM	88·32	88·97	88·79	89·73	89·48	89·81
Crude protein	20·05	20·82	19·66	23·96	23·22	24·30
Ether extract	4·80	5·04	5·07	6·80	7·04	6·96
Neutral-detergent fibre	11·01	11·49	11·35	18·34	19·36	19·80
Ash	6·24	5·93	5·79	6·28	7·09	7·12
Titanium dioxide	0·39	0·42	0·46	0·48	0·51	0·47

* Two basal diets: maize–soyabean meal (MSBM) or MSBM plus 30 % distillers dried grains with solubles (MSBM + DDGS) with three levels of viscosity (i.e. CEL, non-viscous cellulose; MCMC, medium-viscous carboxymethylcellulose; HCMC, high-viscous carboxymethylcellulose,).

† The premix provided the following per kg of complete diet: vitamin A, 3600 μg retinol equivalents; vitamin D_3_, 62.5 μg; vitamin E, 30 μg DL-α-tocopherylacetate; vitamin K_3_, 3 mg; vitamin B_12_, 0.012 mg; riboflavin, 4 mg; niacin, 40 mg; pantothenic acid, 15 mg; choline chloride, 400 mg; folic acid, 0.7 mg; thiamin, 1.5 mg; pyridoxine, 3 mg; biotin, 0.1 mg; Zn, 105 mg; Mn, 22 mg; Fe, 84 mg; Cu, 10 mg; I, 0.50 mg; Se, 0.35 mg.

‡ CEL = cellulose (Ticalose^®^ 100 cellulose powder).

§ MCMC = medium-viscosity CMC (Ticalose^®^ CMC 6000).

|| HCMC = high-viscosity CMC (Ticalose^®^ CMC 15000).

### Sample collection

Pigs were fed their respective experimental diets for 29 d. Ileal digesta samples were collected for 8 h on day 27 and day 28, starting at 08.00 h until 16.00 h and using a 225 ml plastic bag attached to the cannula barrel using a cable tie. Bags were removed every 30 min or whenever full. Ileal digesta samples from the 2-d collection were pooled into 1 litre wide-mouth bottles and a 50 ml digesta sub-sample was used for determining viscosity. Ileal digesta samples were stored at −20ºC immediately until further analysis.

On day 29, pigs were weighed and euthanised by captive bolt followed by exsanguination, and the gastrointestinal tract was removed immediately. Digesta samples (5 ml) from the jejunum and ileum were collected, snap-frozen in liquid N_2_ and stored at −80°C for the analysis of enzymatic activity. Tissue segments (2 cm in length) of jejunum (1 m distal to the pyloric sphincter) and ileum (15 cm proximal to the ileocecal valve) were collected and fixed overnight in Carnoy’s solution for histological evaluation. About 2 g of jejunal samples was collected, snap-frozen in liquid N_2_ and then stored at −80°C until analysis for gene expression. All samples were identified with numbers and identifiers were only associated with the respective treatment at the time of statistical analysis.

### Chemical analysis

Pooled frozen ileal digesta samples were thawed and kept on ice, mixed thoroughly, sub-sampled and lyophilised. Dried digesta samples were ground to pass a 1 mm screen. Diet and digesta samples were analysed following AOAC official method (AOAC, 2012) for crude protein (CP, method 990.03) and diethyl ether extract (EE, method 996.01) at the University of Missouri Experiment Station and Chemical Laboratories. Moisture (method 930.15), ash (method 942·05) and NDF (Ankom NDF method A200) were analysed at the University of Minnesota. The gross energy in all samples was determined using a bomb calorimeter (model 6400; Parr Instrument Co.). The concentration of TiO_2_ in diets and ileal digesta was analysed as described by Myer *et al.*^(20)^.

### Viscosity measurement

Ileal digesta samples were thawed on ice prior to viscosity measurement using an Advanced Rheometric Expansion System with stress-controlled Rheometer (TA Instruments). The measurement was according to the method described by Shelat *et al.* with minor modifications^(21)^. A vane rotor with grooved cup was used for whole digesta viscosity measurement. Digesta samples were loaded into a temperature-controlled cup (39 °C) and pre-warmed for 5 min before measurement. The peak hold test was performed at 0·1 per s shear rate for 2 min. The steady shear measurements were performed for shear rates ranging from 0·1 to 100 per s. All measurements were performed at 39 °C to approximate the body temperature of pigs. For measuring the viscosity of the digesta supernatant, supernatants were obtained after centrifugation at 3500 ***g*** for 10 min. Approximately 15 ml of digesta supernatant was loaded into the rheometric system. Steady shear ﬂow measurements were conducted using a concentric cylinder geometry with a cone DIN rotor (30 mm diameter) at a gap of 500 µm with shear rates ranging from 0·1 to 100per s. The viscosity of whole digesta exhibited non-Newtonian flow behaviour and therefore was fitted using the power-law model below according to Holdsworth^(22)^ *η* = K × *γ*^n−1^, where *η* is the viscosity (mPa·s), K is the consistency constant, *γ* is the shear rate and *n* is the power-law index or flow behaviour index.

### Histological analysis and goblet cell quantitation

Intestinal tissue samples were fixed, trimmed, dehydrated and embedded in paraffin. Slides with 5 µm tissue sections were stained with periodic acid-Schiff with Alcian blue (Newcomer Supply) following the manufacturer’s instructions. Histological analysis was performed as previously described by Saqui-Salces *et al.*^(7)^. Well-oriented villi and crypts were measured in ten randomly chosen fields per slide at 100× magnification and tissue area occupied by goblet cells was measured in five randomly chosen fields per slide at 200× magnification under light microscopy (Olympus BX53) using the CellSense image software (Olympus). Data presented are the means of the average of the fields per pigs in each treatment.

### Digestive enzyme activity analysis

Jejunal and ileal digesta were thawed on ice. Digesta (1 g) was extracted by adding 2 ml ice-cold PBS (1×), vortex and then subjected to centrifugation (3000 ***g*** for 15 min). *α*-Amylase, trypsin and chymotrypsin activities were determined using commercial kits^(23)^ (Biovision K711-100, K771-100 and K352-100) following the manufacturer’s instructions.

### Gene expression analysis

Total RNA from jejunal tissue was isolated using the RNeasy Plus Universal Mini Kit (Qiagen) following the manufacturer’s instructions. Total RNA was quantified using a NanoDrop 2000 instrument (Thermo Scientific), and 500 ng of RNA was reverse transcribed using the High Capacity cDNA reverse Transcription Kit (Applied Biosystems). The expression of genes of interest was determined using Power SYBR Green PCR Master Mix (Applied Biosystems) in a QuantStudio 3 real-time PCR system (Applied Biosystems). The PCR conditions were initial activation at 95 °C for 10 min, followed by 40 cycles of 95°C for 15 s, denaturation and annealing at 60 °C for 60 s. The primer sequences are shown in online Supplementary Table S1. Relative gene expression was calculated using the primer efficiency values as described by Pfaffl^(24)^. The target gene expression was normalised to the gene expression of the reference gene, *GAPDH*, and the target gene expression of each sample was normalised to the mean of the control group (CSMB + CEL).

### Apparent ileal digestibility and its calculation

The apparent ileal digestibility (AID) of nutrients was measured using TiO_2_ as an indicator to normalise feed intake. The TiO_2_, CP, EE, ash and DM in diets and digesta samples were determined as previously described in the ‘Chemical analysis’ section. The AID of nutrients was calculated based on the equation: AID, % = [1 − (N digesta/N diet) × (M diet/M digesta)] × 100, where N digesta and N diet are the nutrient concentrations (g/kg) in digesta and diet DM, respectively, and M diet and M digesta are the TiO_2_ concentrations (g/kg) in diet and digesta DM, respectively.

### Statistical analysis

Statistical power and sample size analyses were performed using SAS (SAS Institute Inc.). *A priori* sample size calculation indicated that a minimal number of six pigs per group were required to achieve significance in a two-way ANOVA. Considering nutrient digestibility of DM as a primary outcome of the present study, retrospective power analysis showed a power of 0·85 was reached on the main effect of fibre and viscosity using GLMPOWER procedure of SAS based on *α* level of 0·05.

Data were analysed using the MIXED procedure with each pig as the experimental unit. The model included fibre content of basal diets (i.e. MSBM *v*. MSBM + DDGS), viscosity (i.e. CEL, MCMC, HCMC) and their two-way interaction as fixed effects, and block was considered as random effect. Treatment means were calculated using the LSMEANS statement and least-square means were compared using the PDIFF statement with Tukey–Kramer adjustment. Linear and quadratic polynomial contrasts were performed to evaluate dose response to increase viscosity on BW, average daily gain (ADG) and nutrient digestibility. Polynomial contrast coefficients were adjusted for unequally spaced treatments using the interactive matrix language (IML) procedure. Pearson correlation analysis was carried out to determine the relationship between viscosity, digestibility, enzymatic activities and nutrient transporters. The power-law model parameters for whole digesta viscosity (*k* and *n*) were estimated for each pig using PROC NLIN. Differences were considered statistically significant if *P* < 0·05 and a trend if *P* < 0·1.

## Results

### General observations

Pigs consumed all of their assigned rations and no signs of diarrhoea were observed throughout the study. General growth performance and health of pigs fed the experimental diets were normal for pigs of this age, feeding regimen and cannulation conditions. After ileal digesta collection on day 28, three pigs were removed from the study (one in MSBM + CEL, one in MSBM + MCMC and one in DDGS + MCMC) due to undefined health issues.

### Viscosity of ileal digesta and growth performance

Inclusion of CMC increased the viscosity of whole digesta (*P* = 0·003) and digesta supernatant (*P* < 0·0001) in the ileum, while increased fibre content from adding DDGS to diets did not affect digesta viscosity (Table 2). The viscosity of whole digesta fitted a power-law index model. There were no differences for the consistency constant among the treatments, indicating that the viscosity of whole digesta was driven by the power-law index when shear rate was fixed. Digesta viscosity resulting from inclusion of CMC (MCMC or HCMC) in the diets had greater (*P* = 0·003) power-law index than that of digesta of the CEL group regardless of fibre content, indicating that digesta from CMC-fed pigs showed thicker flow behaviour than digesta from CEL-fed pigs. Similarly, inclusion of CMC in diets resulted in greater viscosity of digesta supernatant (*P* < 0·0001) than CEL group regardless of fibre content. Inclusion of HCMC had higher digesta viscosity (*P* = 0·04) than MCMC, but fibre content did not interact with viscosity (i.e. the addition of CMC) to alter digesta viscosity. These results provided a proof of concept that viscous CMC, but not DDGS fibre, increased the viscosity of whole digesta and digesta supernatant in the small intestine of pigs. Thus, our design allowed evaluating the two variables – fibre content and viscosity.

**Table 2.** Rheological characteristics of ileal digesta from pigs fed experimental diets containing CMC and DDGS*

Fibre content	MSBM			DDGS			SEM†	*P* ‡		
Viscosity	CEL	MCMC	HCMC	CEL	MCMC	HCMC		F	V	F × V
Whole digesta§										
K	5·10	4·71	5·03	4·05	3·52	7·48	1·47	0·95	0·32	0·38
*n*	0·19^b^	0·37^a^	0·39^a^	0·21^b^	0·18^a,b^	0·40^a^	0·05	0·24	0·003	0·11
Digesta supernatant, mPa·s										
Viscosity	1·28^c^	6·98^b^	9·24^a^	1·53^c^	6·97^b^	11·25^a^	1·25	0·48	<0·0001	0·69

a,b,c Mean values within a row with unlike superscript letters were significantly different (*P* < 0.05).

* Two basal diets: maize–soyabean meal (MSBM) or MSBM plus 30 % distillers dried grains with solubles (MSBM + DDGS) with three levels of viscosity (i.e. CEL, non-viscous cellulose; MCMC, medium-viscous carboxymethylcellulose; HCMC, high-viscous carboxymethylcellulose).

† Standard error of the mean.

‡ Factorial arrangement of treatments fibre (F) and viscosity (V) main effects with eighteen and twelve observations, respectively, and fibre and viscosity interaction (F × V) with six observations per treatment.

§ The dynamic whole digesta viscosity was fitted a power-law model according to Holdsworth^(23)^: *η* = K × *γ*^*n*−1^, where *η* is the viscosity, K is the consistency constant, *γ* is the shear rate and *n* is the power-law index.

For pig growth performance, ADG and final BW declined quadratically (*P* = 0·01) with increasing viscosity (Table 3). Inclusion of MCMC and HCMC in the diets decreased (*P* = 0·0002) ADG by about 200 and 165 g, respectively, compared with CEL regardless of fibre content. No interactions of viscosity and fibre content were observed. Pigs fed diets containing MCMC and HCMC had 14 and 9 % lower (*P* = 0·0004) final BW than those fed CEL because ADG directly affects final BW. Because pigs were fed two times daily with the amount of feed equivalent to three times the maintenance energy requirement, the decreased growth performance is most likely attributed to reduced digestibility of nutrients and energy.

**Table 3.** Body weight and average daily gain in pigs fed diets differing in fibre content and viscosity*

Fibre content	MSBM			DDGS			SEM	*P* †				
Viscosity	CEL	MCMC	HCMC	CEL	MCMC	HCMC		F	V	F × V	Linear	Quadratic
Initial BW, kg	26·68	26·16	26·40	26·33	26·78	26·92	1·73	0·46	0·08	0·47	NA	NA
Final BW, kg	56·44^a^	48·67^b^	49·29^b^	56·34^a^	48·29^b^	52·30^b^	2·45	0·38	0·0004	0·59	0·0006	0·01
ADG, kg	0·84^a^	0·61^b^	0·64^b^	0·83^a^	0·62^b^	0·70^b^	0·04	0·50	0·0002	0·68	0·0003	0·01

a,b Mean values within a row with unlike superscript letters were significantly different (*P* < 0.05).

* Two basal diets: maize–soyabean meal (MSBM) or MSBM plus 30 % distillers dried grains with solubles (MSBM + DDGS) with three levels of viscosity (i.e. CEL, non-viscous cellulose; MCMC, medium-viscous carboxymethylcellulose; HCMC, high-viscous carboxymethylcellulose).

† Factorial arrangement of treatments fibre (F) and viscosity (V) main effects with eighteen and twelve observations, respectively, and fibre and viscosity interaction (F × V) with six observations per treatment. Polynomial (linear and quadratic) contrasts were used to determine the effect of viscosity.

### Apparent ileal digestibility

**Fig. 1.**
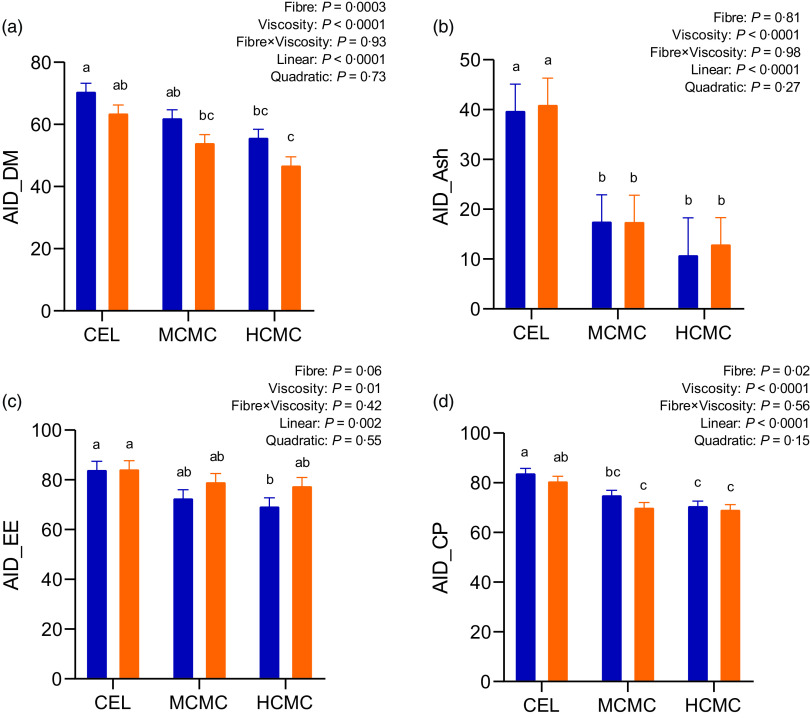
Apparent ileal digestibility (AID) of DM (A), ash (B), ether extract (C) and crude protein (D) in pigs fed diets differing in fibre content and viscosity. Bars represent LS means ± sem, *n* 6. Polynomial (linear and quadratic) contrasts were used to determine the effect of viscosity. MSBM, maize–soyabean meal; DDGS, MSBM plus 30 % maize distillers dried grains with solubles; CEL, non-viscous cellulose; MCMC, medium-viscous carboxymethylcellulose; HCMC, high-viscous carboxymethylcellulose. ^a,b,c^Different letters indicate values are different (*P* < 0·05). 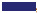, MSBM; 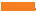, DDGS.

As anticipated, both fibre content and viscosity negatively affected nutrient digestibility (Fig. 1). Increased fibre content from adding DDGS (MSBM + DDGS diets) decreased AID of DM (*P* = 0·0003), EE (*P* = 0·06) and CP (*P* = 0·02) compared with MSBM diets. Pigs fed diets containing MCMC and HCMC had lower AID of DM (*P* < 0·0001), ash (*P* < 0·0001), EE (*P* = 0·01) and CP (*P* < 0·0001) compared with pigs fed diets containing CEL regardless of fibre content, and no differences were observed between MCMC and HCMC treatments. AID of DM, ash, EE and CP decreased linearly (*P* < 0·0001, *P* < 0·0001, *P* = 0·002 and *P* < 0·0001) with increasing viscosity from adding MCMC and HCMC. These results suggested that viscosity and fibre content independently decreased nutrient digestibility in the small intestine.

### Intestinal epithelial responses to dietary fibre and viscosity

Feeding diets with increased viscosity resulted in longer villi (*P* = 0·06), deeper crypts (*P* = 0·02) in the jejunum and deeper crypts (*P* = 0·09) in the ileum compared with those in the CEL group (Table 4). However, feeding diets with increased fibre content did not affect tissue morphology of the small intestine. The goblet cell area in the ileum of pigs fed diets with CMC inclusion was 52 % greater (*P* = 0·004) compared with the ileum of pig in the CEL group. Pigs fed MCMC and HCMC had similar goblet cell area in the ileum. As for morphology, increased fibre content had no impact on goblet cell area in the small intestine, nor did it interact with viscosity to modify morphology and goblet cells area. These results suggest that viscosity, rather than the amount of DF, is a primary cause of the adaptation of intestinal epithelium.

**Table 4.** Intestinal morphology and goblet cell area in pigs fed diets differing in fibre content and viscosity*

Fibre content	MSBM			DDGS			SEM†	*P* ‡		
Viscosity	CEL	MCMC	HCMC	CEL	MCMC	HCMC		F	V	F × V
Jejunum										
Villus height, µm	312·76	326·73	384·02	333·06	340·53	375·32	24·73	0·68	0·06	0·82
Crypt depth, µm	99·51^b^	99·73^b^	127·09^a^	109·99^b^	101·16^b^	127·85^a^	9·64	0·60	0·02	0·86
Villus:crypt ratio	3·37	3·44	3·25	3·24	3·50	3·19	0·13	0·84	0·66	0·94
Goblet cell area, %§	3·55	4·17	4·03	3·39	3·49	3·88	0·52	0·45	0·64	0·85
Ileum										
Villus height, µm	307·69	333·29	376·50	351·26	358·18	367·81	20·93	0·25	0·12	0·42
Crypt depth, µm	209·44	257·15	267·66	238·24	250·60	244·11	17·47	0·97	0·09	0·23
Villus:crypt ratio	1·53	1·34	1·47	1·54	1·49	1·55	0·08	0·23	0·35	0·69
Goblet cell area, %§	7·59^b^	12·84^a^	12·26^a^	8·08^b^	11·02^a^	11·68^a^	1·22	0·53	0·004	0·66

a,b Mean values within a row with unlike superscript letters were significantly different (*P* < 0.05).

* Two basal diets: maize–soyabean meal (MSBM) or MSBM plus 30 % distillers dried grains with solubles (MSBM + DDGS) with three levels of viscosity (i.e. CEL, non-viscous cellulose; MCMC, medium-viscous carboxymethylcellulose; HCMC, high-viscous carboxymethylcellulose).

† Standard error of the mean.

‡ Factorial arrangement of treatments fibre (F), viscosity (V) main effects with eighteen and twelve, observations respectively, and fibre and viscosity interaction (F × V) with six observations per treatment.

§ Goblet cells area was defined as area of positive cells for PAS-AB staining/mucosal area × 100.

The gene expression of monosaccharides and amino acid transporters in the jejunum were not influenced by viscosity, fibre content or their interaction (Table 5).

**Table 5.** Relative mRNA expressions of nutrient transporters in the jejunum of pigs fed diets differing in fibre content and viscosity*

Fibre content	CSB			DDGS			SEM†	*P* ‡		
Viscosity	CEL	MCMC	HCMC	CEL	MCMC	HCMC		F	V	F × V
Sugar transporters§										
*GLUT2*	1·00	1·03	1·02	0·92	0·96	0·98	0·05	0·17	0·74	0·94
*GLUT5*	0·99	1·00	0·96	0·97	0·96	0·88	0·09	0·50	0·66	0·92
*SGLT1*	1·00	1·04	0·98	0·90	1·01	0·98	0·07	0·41	0·56	0·70
Amino acid transporters||										
*TAT1*	1·00	1·04	0·98	0·90	1·00	0·99	0·08	0·48	0·63	0·74
*b0*,+*AT*	1·00	1·01	1·00	0·86	1·00	0·95	0·08	0·34	0·65	0·75
*B0AT1*	1·00	1·05	0·98	0·83	1·03	0·98	0·10	0·48	0·53	0·07
*LAT2*	1·00	1·05	0·97	0·91	1·01	1·00	0·08	0·66	0·65	0·74
*CAT1*	1·00	1·04	0·97	0·89	1·00	0·97	0·07	0·39	0·53	0·70
*ASCT2*	1·00	1·12	0·96	0·88	1·01	0·97	0·09	0·32	0·33	0·71
*PEPT1*	1·00	1·11	1·03	0·90	1·06	1·03	0·07	0·43	0·21	0·77

* Two basal diets: maize–soyabean meal (MSBM) or MSBM plus 30 % distillers dried grains with solubles (MSBM + DDGS) with three levels of viscosity (i.e. CEL, non-viscous cellulose; MCMC, medium-viscosity carboxymethylcellulose; HCMC, high-viscosity carboxymethylcellulose).

† Standard error of the mean.

‡ Factorial arrangement of treatments fibre (F), viscosity (V) main effects with eighteen and twelve observations, respectively, and fibre and viscosity interaction (F × V) with six observations per treatment.

§ *GLUT2* = solute carrier family 2 member 2 (*SLC2A2*), *GLUT5* = solute carrier family 2 member 5 (*SLC2A5*), *SGLT1* = sodium-dependent glucose cotransporter 1 (*SLC5A1*).

|| *ASCT2*, alanine, serine, cysteine transporter 2 (*SLC1A5*); *AT1*, T-type amino acid transporter 1 (*SLC16A10*); b0,^+^AT, b(0,^+^)-type amino acid transporter 1 (*SLC7A9*); *B0AT1*, B(0,+)-type amino acid transporter 1 (*SLC6A19*); *CAT1*, cationic amino acid transporter 1 (*SLC7A1*); *LAT2*, large neutral amino acids transporter small subunit 2 (*SLC7A8*); *PEPT1*, peptide transporter solute carrier family 15 member 1 (*SLC15A1*).

### Digestive enzymes activities

**Fig. 2.**
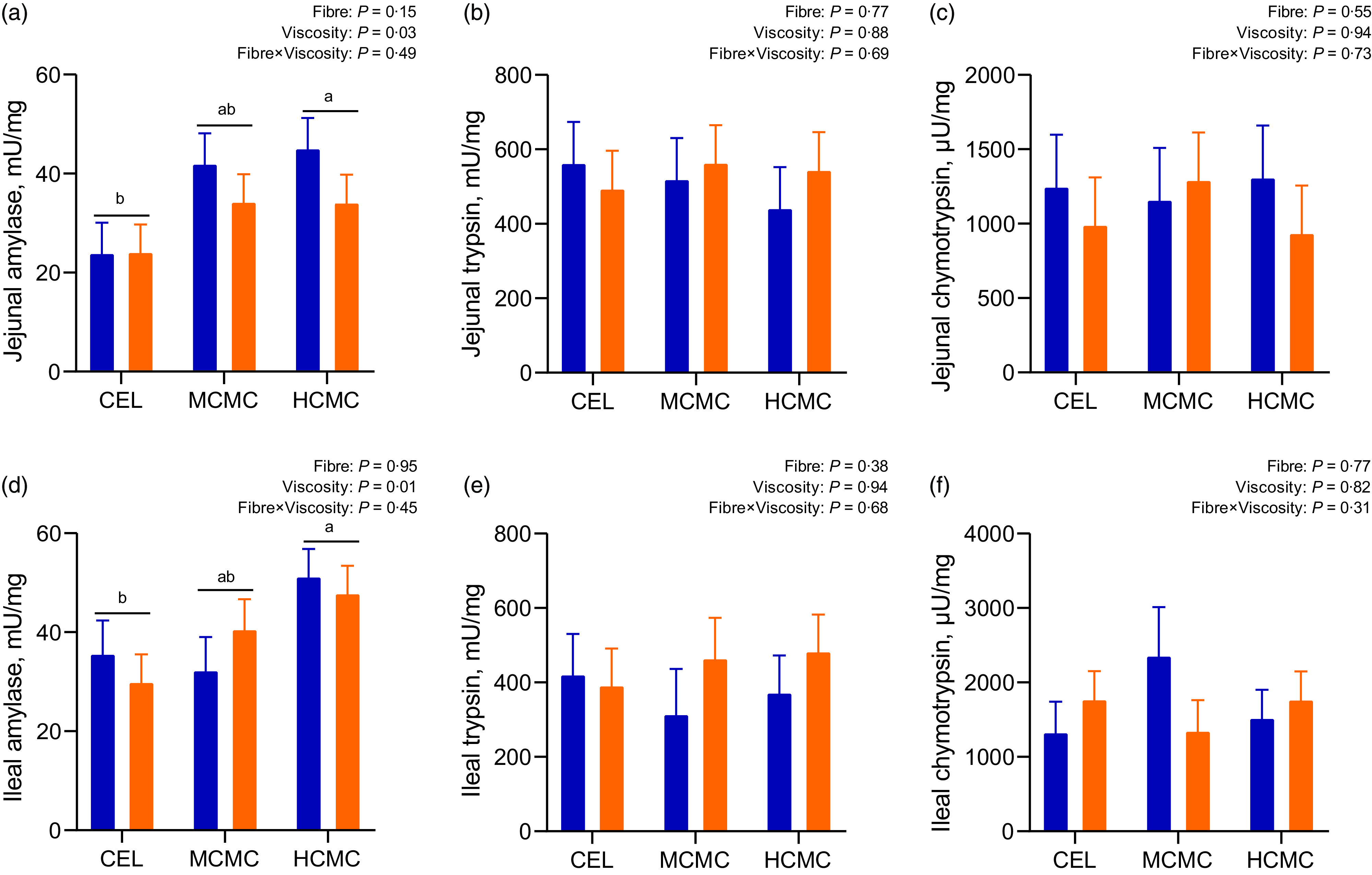
Enzymatic activities in the intestinal digesta of pigs fed diets differing in fibre content and viscosity. Bars represent LS means ± sem, *n* 6. MSBM, maize–soyabean meal; DDGS, MSBM plus 30 % maize distillers dried grains with solubles; CEL, non-viscous cellulose; MCMC, medium-viscous carboxymethylcellulose; HCMC =high-viscous carboxymethylcellulose. ^a,b^Different letters indicate differences (*P* < 0·05) among viscosity treatments regardless of fibre content. 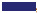, MSBM; 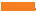, DDGS.

Increased viscosity from dietary inclusion of MCMC and HCMC increased amylase activities by 40 and 46 % in the jejunal digesta (Fig. 2(A); *P* = 0·03), and by 11 and 50 % in the ileal digesta, respectively (Fig. 2(D); *P* = 0·01) compared with enzymatic activities in the digesta of the CEL group (Fig. 2). No differences were observed for activity of trypsin and chymotrypsin in the jejunum or ileum. No interactions between viscosity and fibre content were observed for digestive enzymatic activities in the digesta of jejunum and ileum.

### Correlation analysis for viscosity effect

**Fig. 3.**
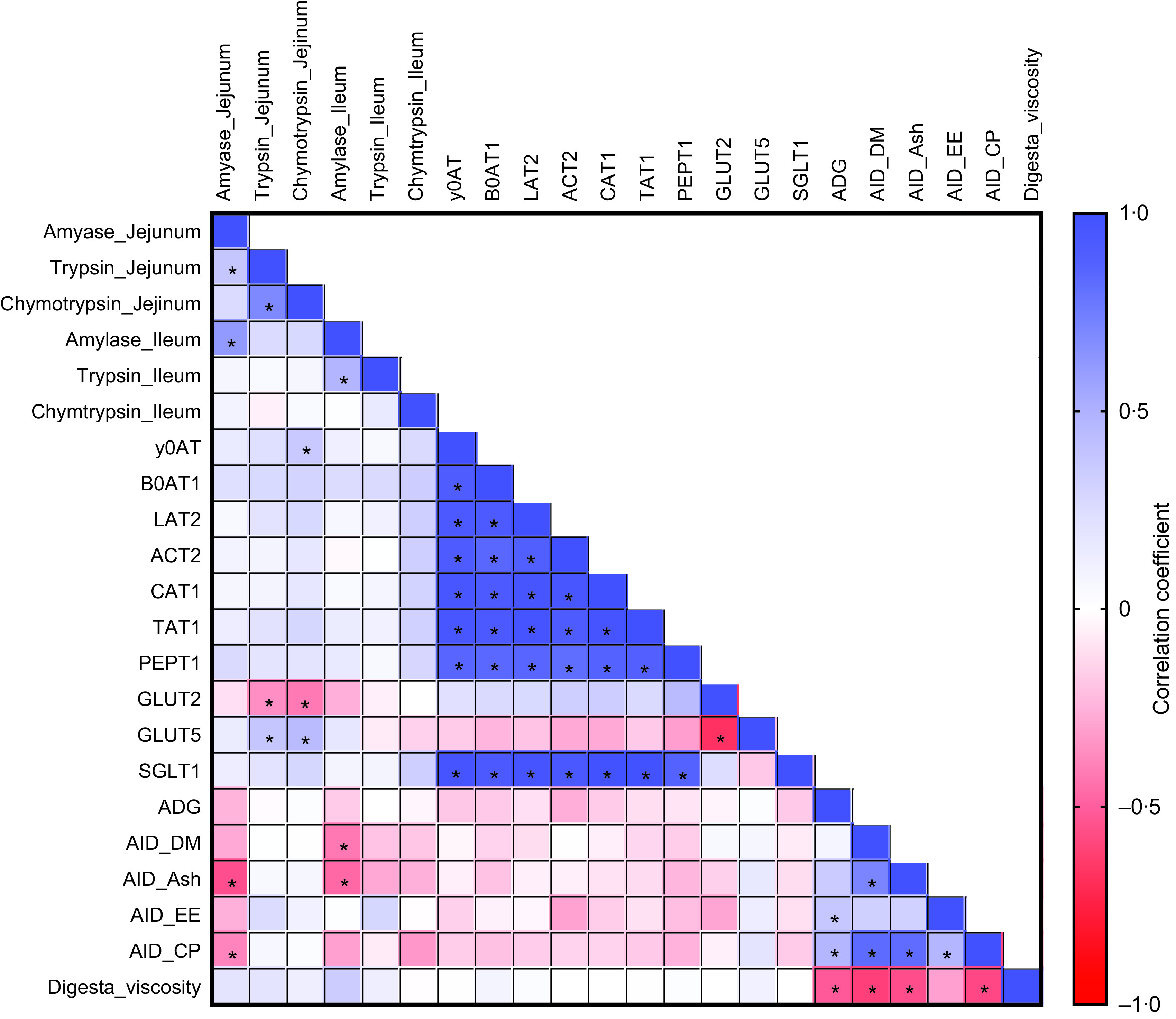
Pearson correlation analysis is expressed in a heatmap. *P* < 0·05 is indicated with *.

To further evaluate the relationships between the parameters measured in the study, we performed a correlation analysis. Several significant correlations were observed among saccharide and amino acid transporters, nutrient digestibility, ADG and digesta viscosity (Fig. 3). ADG was positively correlated with AID of EE (*r* 0·37; *P* = 0·03) and CP (*r* 0·47; *P* = 0·006). Digesta viscosity was negatively correlated with AID of DM (*r* −0·60; *P* < 0·001), ash (*r* −0·56; *P* = 0·001), CP (*r* −0·57; *P* < 0·001) and ADG (*r* −0·52; *P* = 0·002). These correlations suggest a positive association between nutrient digestibility and growth performance and a negative association between viscosity and nutrient digestibility.

## Discussion

In the present study, increasing DF content and viscosity were achieved by adding DDGS and CMC. Dietary inclusion of CMC is a practical way to manipulate digesta viscosity without changes on other physical–chemical properties of DF as demonstrated in poultry^(16)^ and swine studies^(13,17,25)^. Regarding digesta viscosity, the present study showed that dietary inclusion of CMC increased viscosity of whole digesta and digesta supernatant. Many studies have only measured viscosity in the digesta supernatant^(13,16,17,25)^. However, the rheological behaviour of the digesta supernatant is not representative of the whole digesta^(26)^. Likewise, the majority of experiments that have evaluated the effects of fibre or viscosity on intestinal function were conducted using soluble DF sources^(13,23,27)^. This presents a disadvantage because most common DF sources (e.g. whole-wheat flour, cereal grain bran, vegetables, DDGS, wheat middlings, rice bran) contain high quantities of insoluble DF. Diets and feedstuffs are a mixture of soluble and insoluble NSP that can be degraded throughout the gastrointestinal tract, changing the viscoelastic properties of the digesta^(28)^. Thus, analysing the effects of insoluble DF on viscosity and intestinal responses is needed. In our study, the addition of DDGS increased insoluble DF by 7 % but this amount did not affect digesta viscosity as CMC addition did, suggesting that fibre solubility may be a stronger determinant of digesta rheological properties than the amount of insoluble fibre.

Although the use of ileal-cannulated pigs in digestibility trials is not an ideal model to assess growth performance, we observed substantial decrease in ADG and final BW of pigs fed diets with increased viscosity even when pigs consumed the same amount of feed and metabolisable energy among dietary treatments. Similar observations have been reported previously in both broilers^(29–31)^ and pigs^(13,17,32)^. We also observed that digesta viscosity was negatively correlated with ADG, which is in agreement with observations made in broilers^(30)^ and pigs^(13)^. Increased viscosity impairs nutrient digestibility and energy utilisation that could be responsible for reduction of ADG and final BW^(16,29,32)^. Our results indicate that increased digesta viscosity is detrimental to growth performance.

A reduction in nutrient digestibility indicates less nutritional value and net nutrient supply from the diet that could be used for body functions. Feeding high-fibre diets can decrease nutrient digestibility in humans^(9)^ and pigs^(4,10)^. In agreement with previous studies, our results showed that pigs consuming increased amount of DF had decreased AID of DM and CP compared with pigs fed MSBM diets. Several mechanisms contributing to this negative effect of DF on nutrient utilisation have been proposed. Among them are reduced enzymatic activity because of complex starch-protein-cell wall matrix that may limit nutrient digestion^(4,33,34)^, decreased retention time because of insoluble DF leading to less time for digestion^(4)^ and the formation of a gel-viscous barrier limiting enzymatic action^(4,33,35)^. Also, increasing DF content and its associated viscosity would likely increase specific endogenous losses, including sloughed cells, mucins and intestinal and pancreatic secretions^(36)^, thereby decreasing apparent nutrient digestibility^(16)^. However, the approaches used in the present study did not allow the measurement of these losses.

Although most studies in broilers^(16,29,31,37)^ and pigs^(32,38–40)^ have reported negative effects of viscosity on nutrient digestibility, other studies have reported no detrimental effects of viscosity on nutrient digestibility in pigs^(13,25)^. This discrepancy may result from the diet composition. Semi-purified diets are highly digestible compared with cereal-based diets and have been used in the studies that reported no negative effect of viscosity on nutrient digestibility^(13,25)^. It is important to note that the lack of a standard procedure measuring digesta viscosity limits our capacity for analysing and concluding from the literature because of methods and value variations. Therefore, a consensus is needed for the procedure to measure digesta viscosity in the near future. We proposed the methods used in the present study to be used as standard, as they consider the complex composition and rheology of digesta.

The effect of DF on goblet cells and mucin production has been reported in murine^(27,41)^, chicken^(31)^ and pig models^(6,7,25,42)^. However, it is unclear whether this response is directly related to DF content or to its rheological properties. In the present study, an increase in goblet cells was in accordance with previous publications^(25,27)^. More importantly, we found that viscosity but not fibre content induced this change in the ileum, suggesting that the rheological property of the digesta is the main driver for goblet cell expansion and mucin production in the distal small intestine. Although mucin produced from goblet cells has a protective and beneficial effect for the host, mucin is considered as a non-dietary antagonist because when it is in excess, nutrient digestibility decreases^(36)^ and represents energy and nutrient loses for production animals^(43)^. The number of goblet cells and amounts of mucins secretion that provide protection without resulting in production loses is still undetermined.

Results from a recent study revealed that gene expression of nutrient transporters and enzymes in the jejunum are involved in the changes of nutrient digestibility of growing pigs^(44)^. A few studies have shown that feeding DF or certain NSP increases gene expression of intestinal nutrient transporters, mainly *SGLT1, GLUT2, GLUT5 and PEPT1*^(7,45,46)^, suggesting an adaptation in response to nutrient availability in the gut. In the present study, several amino acid transporters in addition to monosaccharide and peptide transporters were analysed, but neither fibre content nor viscosity affected the gene expression of nutrient transporters in the jejunum. This discrepancy may be due to the concentration of DF used in the previous studies, the age of pigs and the nutritional composition and the methods of measurement of DF of the diets.

Digestibility may also be determined by changes in digestive enzyme secretion. Our results suggested that activities of amylase are affected by viscosity rather than DF content, which is in agreement with the finding that soluble DF intake decreases macronutrient digestion by reducing digestive enzyme activities^(23,47,48)^. Nevertheless, previous *in vitro* and *in vivo* studies have shown different responses on the effect of viscosity on digestive enzyme activities. Increasing viscosity by NSP in starch-digestive enzyme suspensions depressed glucose diffusion in a dialysis tube, suggesting the suppression of enzymatic hydrolysis and nutrient diffusion by viscosity^(49)^. Isaksson *et al.* demonstrated that increased viscosity by adding pectin to jejunal juice of humans reduced activities of trypsin and amylase^(47)^. In contrast, the activities of amylase, protease and lipase in the pancreas and on pancreatic-biliary secretion were increased in rats fed viscous guar gum for 14 d compared with control^(50)^, and increased amylase activity has been reported in pigs fed pectin for 21 d compared with those fed control diets^(23)^. Because of the very different models and compounds used in the literature, to define the effect of chyme viscosity on digestive enzymatic activity requires further research.

Some limitations to the present study merit consideration. The experimental procedures (i.e. cannulation, housed individually and fixed amount of daily feed) used in the study deviate from the norm for raising pigs. Experimental conditions and the number of animals limit our capability to make conclusions on growth performance. Other factors such as feed intake and solubility of nutrients influence retention time of solids and liquids in the gastrointestinal tract, thereby changing the kinetics of nutrient flow^(51)^. Although soluble DF and diet viscosity might influence retention time^(52)^, the role of digesta viscosity on retention time cannot be discerned in the present study. The impact of retention time on nutrient digestibility of complex diets requires further evaluation. The viscosities achieved for HCMC and MCMC diets, and the corresponding AID, were not as different as expected. This limited our capability to estimate the broader effects of viscosity on nutrient digestibility. Because of the number of treatments and range of non-equally spaced viscosity, inferences on dose–response effects are not conspicuous, although the linear and quadratic responses shed some light on dose effects. Finally, we used CMC, a soluble, non-fermentable fibre, to manipulate viscosity. However, the effects observed in the present study may not apply to all soluble DF sources because not all soluble DF are viscous, such as inulin, fructooligosaccharides and wheat dextrin^(1)^.

In conclusion, the present results support the hypothesis that viscosity has a significant impact on digestive function. Although increased DF content and viscosity independently decreased nutrient digestibility, the content of DF fed had no effect on ADG, final BW, intestinal morphology, goblet cell area and digestive enzymatic activities. In contrast, increased digesta viscosity regardless of fibre content resulted in decreased ADG and final BW, deeper crypts, and greater goblet cell area and greater amylase activity, suggesting that viscosity is the dominant factor that affects intestinal digestive physiology. Results from our study emphasise the need for considering the variable viscosity properties of high-fibre ingredients in particular ingredients high in viscous DF, used in human food and formulating animal diets to more closely predict effects on nutrient digestibility and improve DF utilisation.

## Supplementary material

For supplementary material accompanying this paper visit https://doi.org/10.1017/S0007114521000866. click here to view supplementary material
