# Supplementary material for: Decreased nutrient digestibility due to viscosity is independent of the amount of dietary fibre fed to growing pigs
Source: Br J Nutr. 2021 Mar 12;127(2):177–87. doi: 10.1017/S0007114521000866 (PMC8756099; doi:10.1017/S0007114521000866)
Supplement: Supplementary file 1 [file S0007114521000866sup001.docx]

**Table S1.** Genes of interest and primer sequences used in this study

| Gene^1^ | Forward | Reverse |
| --- | --- | --- |
| *GLUT2* | TTTTGGGTGTTCCGCTGGAT | GAGGCTAGCAGATGCCGTAG |
| *GLUT5* | TGTGTGGCTCCTGGTAACAC | TCGGCCATGTTCGATTCCTT |
| *SGLT1* | CCCAAATCAGAGCATTCCATTCA | AAGTATGGTGTGGTGGCCGGTT |
| *TAT1* | GGATTTCTGCTCGGATTCAT | AACAAAGGACAACACCTCCAA |
| *b0,^+^AT* | GAACCCAAGACCACAAATC | ACCCAGTGTCGCAAGAAT |
| *B0AT1* | ACAACAACTGCGAGAAGGACTC | GCAGGTCAAACCCGTTGATAAG |
| *LAT2* | CGGAGACTGGTTCTGGAGAG | AGTTGACCCATGTGAGGAGC |
| *CAT1* | GAGCAAGACCAAACTCTCCTTC | AGCCTATCAGCATCCACACTG |
| *ASCT2* | GATTGTGGAGATGGAGGATGTGG | TGCGAGTGAAGAGGAAGTAGATGAGA |
| *PEPT1* | TTGTGGCTCTGTGCTACCTG | TCCGTTGTGGTCGAAGTCTG |
| *GAPDH* | ATCCTGGGCTACACTGAGGAC | AAGTGGTCGTTGAGGGCAATG |

^1^*GLUT2* = solute carrier family 2 member 2 (*SLC2A2*), *GLUT5* = solute carrier family 2 member 5 (*SLC2A5*), *SGLT1* = sodium-dependent glucose cotransporter 1 (*SLC5A1*), *TAT1* = T-type amino acid transporter 1 (*SLC16A10*), b0,^+^AT = b(0,^+^)-type amino acid transporter 1 (*SLC7A9*), *B0AT1* = B(0,+)-type amino acid transporter 1 (*SLC6A19*), *LAT2* = large neutral amino acids transporter small subunit 2 (*SLC7A8*), *CAT1* = cationic amino acid transporter 1 (*SLC7A1*), *ASCT2* = alanine, serine, cysteine transporter 2 (*SLC1A5*), *PEPT1* = peptide transporter solute carrier family 15 member 1 (*SLC15A1*), *GAPDH* = glyceraldehyde 3-phosphate dehydrogenase
